# Natural Frequencies of seiches in Lake Chapala

**DOI:** 10.1038/s41598-019-48319-6

**Published:** 2019-08-14

**Authors:** David Avalos Cueva, Cesar O. Monzón, Anatoliy Filonov, Iryna Tereshchenko, Pedro Limón Covarrubias, José Roberto Galaviz González

**Affiliations:** 1 Department of Civil Engineering and Topography, University of Guadalajara, Guadalajara, Mexico; 2 Department of Projects Engineering, University of Guadalajara, Guadalajara, Mexico; 3 Department of Physics, University of Guadalajara, Guadalajara, Mexico

**Keywords:** Limnology, Hydrology

## Abstract

This research presents an analysis of the frequencies and vibration modes of the structure of the superficial seiches of Lake Chapala using mathematical modelling and measurements. The HAMSOM model was run with wind fields from coastal meteorological stations and for different lake storage levels. The lake water level measurements were carried out in two campaigns in 2003. An analysis of the surface seiches in the lake was performed using the fast Fourier transform method. A spectral analysis of lake water level measurements reveals seiches with periods close to 5.9, 3, 2, and 1.5 h. A comparison with the periods calculated by the HAMSOM model indicates that these periods correspond to superficial seiches of the fundamental longitudinal mode of the lake and the subsequent three modes. The lake has undergone important changes in its storage level over time. Therefore, we modelled it with storage levels from the isobaths 90 to 95 m and found that surface seiche periods decreased to 5.2 h.

## Introduction

In Lake Chapala and other closed or semi-closed water bodies, there are gravitational waves that have greater frequency and lower amplitude, these are generated by different mechanisms that promote natural resonant oscillations known as seiches (own oscillations). Recent studies have shown the existence of long waves, derived from the non-linear interaction of waves and wind waves (infragravity waves). These waves lead to phenomena in surf and coastal zones, such as: water currents, waves shape, crescent bars, beach tops and other forms produced by coastal topography, as sediments by trawling materials^1,2^.

Nevertheless, non-linear interactions and atmospheric processes such as wind and waves, are the commonly factors that induce oscillations in water bodies. Although the physical nature of these fluctuations is very simple, determining the periods and the structure of these waves is very difficult as the configuration of the edge and the depth of the lake varies. Seiches, or free periodic oscillations or normal modes, are generated when the winds suddenly stop over a lake, whereby the inclined surface produced by the accumulation of water tends to establish a static equilibrium. However, due to its inertia, the wave extends beyond this position, thus giving rise to an oscillatory motion around one or several nodal lines^3,4^.

Stationary waves are produced by repetitive reflections, so the shape of these waves is derived from one of the natural oscillation modes of the Lake, which is a function of the size and depth of the basin and the configuration of the coastline^4,5^. The amplitude of the surface seiches depends strongly on the intensity of external forcing, which induced the motion of water basin, producing the natural oscillation period of thereof ^3,6–11^. Thus, stronger atmospheric disturbances usually generate larger seiche oscillations that are part of a broad frequency interval, in which the response of the water body is included, such as its free modes in combination with oscillations produced by forced disturbances, but which are not resonant at other frequencies. When the forced disturbances stop, however, forced seiches decrease abruptly, allowing the natural oscillations to persist longer.

Moreover, since it is known that waves with lower frequencies are generated by higher wind speeds. Hence, when the wave passes from high frequency to low frequency, a change of energy occurs that is related to the growth of the same, this is due to the interactions between waves and their non-linear behavior. Longo, *et al*.^12^ showed that the process varies over time, as the energy dissipates as the root mean square wave height decreases when the wave breaks. Also, they demonstrate through experiments in a laboratory environment that the macro-scales of the turbulence are not sensitive to the passage of wave crests and troughs, and the dominant eddies seem to possess a constant pattern^12^.

Free oscillations or seiches in lakes have been studied over time, and such research has been carried out through the use of vertically integrated models and *in situ* measurements, such as in Lake Geneva^13^, Constance^14,15^, Biwa-ko^16^, Baikal^17^, Flathead^18^, Valunden^10^, Erie^19^, and Michigan^20^. Other research has performed seiche oscillation analyses, Rabinovich and Monserrat^21^ and Longo, *et al*.^1^ such as, and the findings showed powerful atmospheric disturbances do not always generate perceptible seiche oscillations. Moreover, these studies presented novel experimental methods and measurements and showed that water level variations are also caused by the radiation stress balance near the coast, where wave and wind set-up occur.

In Lake Chapala, Filonov^8^ analysed the variations of atmospheric pressure and wind, where he observed that synoptic processes generated pressure increases, and these in turn, always evidenced the production of brusquely amplified seiches. Thus, he concluded that a pressure increase applied in Lake Chapala leads to an obvious slant in the level of a part of the seiche, this pressure generates prolonged free gravity waves, and causes horizontal flows and vertical movements of the water. Additionally, Avalos-Cueva, *et al*.^22^ and Filonov^8^ studied the characteristics of free-surface oscillations (seiches) in the lake; however, their results do not allow for generalization because they were only obtained for one sampling point. Hence, few studies have focused on the frequency, spatial characteristics, and contributions to the formation of extreme variations in lake levels. Although seiches represent the most common type of level oscillation in Lake Chapala, sufficient information is not available on these phenomena.

The purpose of this research was to investigate the main harmonic mode periods of free oscillations (seiches) in Lake Chapala. Periods and amplitudes of the seiches have been estimated via an analysis of the *in situ* time series of water levels. To identify detailed characteristics of the space-time structure of the seiches, mathematical modelling is applied using the HAMSOM model. The modelling is based on the application of the HAMSOM 2D model, which uses the data from the wind field formed by 7 meteorological stations placed at strategic points all over the lake. Likewise, lake-level variability data obtained during three measurement campaigns in 2003 were used. Finally, comparisons of measured seiches with numerical simulations were made.

## Study Area

**Figure 1:**
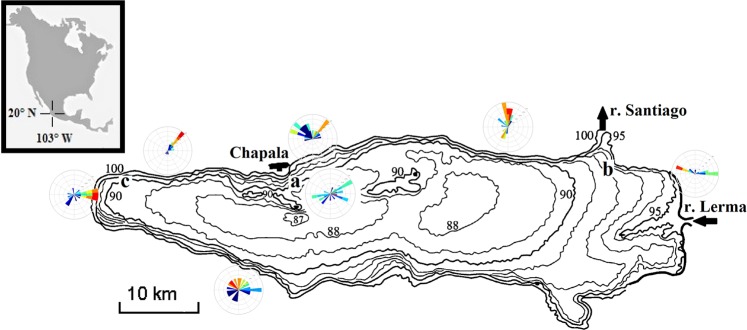
Bathymetric map of Lake Chapala, the value of the level curves are reported according to the local “Bench Mark” (isobaths) scale where the maximum storage water capacity equal to 7,897 Mm^3^ (corresponding to isobaths 97.8 m that is 1523.80 m above sea level). The weather vane represents the distribution and average frequency of the wind direction from the network of the weather stations and measuring sites: (**a**) northeast, (**b**) east and (**c**) west.

Lake Chapala is located in the Transmexicana Volcanic Band, south oriented from the conurbated area of Guadalajara in Jalisco, Mexico (20°15′ North and 103°05′ West) with an altitude of 1,542 meters above sea level (Fig. 1). It is the greatest natural lake in Mexico, covers an area of 1161 km^2^, and stretches over 79 km in length and 28 km in width. On the riverside of the lake, considerable tourism, fishing, industrial, and agricultural activities are observed. Basically, the lake functions as a regulator of the Lerma-Santiago basin. Lake Chapala has as affluent and effluent the Lerma and Santiago Rivers, respectively. It is a shallowlake, characterized by an average and maximum depth between 6 and 11 m, respectively.

Filonov, *et al*.^23^, Avalos-Cueva, *et al*.^22^, Filonov^8^ and Filonov, *et al*.^24^ indicated that the dynamics of the lake is directly affected by the breeze and present winds that are developed daily by the difference in temperature between the surface of the lake and the vegetation cover of the region. Wind and lake breeze speed during the day reaches 8–10 m s^−1 ^^22–24^.

The lake is warm polymictic and only stratifies for a few hours on nonwind days^23^. The lake has a temperate climate, evidencing an annual average temperature of 19.9 °C. On the other hand, the Chapala region experiences rainfall in summer, producing an average precipitation of approximately 750 mm per year, and the surface evaporation is approximately 1400–1600 mm per year. Hence, the deficit is balanced due to the water supply provided by the Lerma River and to the catchment of water from its basin^23–26^.

The lake is turbid (Secchi disk measurement of 0.1–0.7 m), with a great amount of flocculated sediments as a result of wind mixing, sediment resuspension, and Lerma River discharge^27–29^. In recent years, the lake’s storage level has undergone considerable variations; the above is due to the demand in the use of potable water for human consumption, industrial, irrigation, among others. Recently, climatological cycles have been identified that generate important changes in the storage level, among those that most affect are the solar activity and El Niño/Southern Oscillation (ENSO), showing level drops close to 1 m^29^.

## Data and Methods

### Field measurements

Water level data were collected during two campaigns conducted on the lake in 2003. The first campaign was carried out in January, during which two Onset Hobo U20 water-level logger were installed in the lower part of the water column at depths of 1.5 and 2.35 m (these were mounted on a base rectangular with holes allowing free water flow to avoid that these are susceptible to movement and the data record can be disrupted and inaccurate if the logger is knocked over or displaced from the initial position). In the east and northeast areas of the lake (Fig. 1), measurements were recorded with a sampling interval of 15 min to encompass the free oscillations. The submerged sensor has a water-level accuracy of 0.05 percent of full scale (about 0.005-m water depth) and water-level resolution of 0.002-m water depth.

Later in June, the second campaign was carried out, during which a Hobo water level sensor was installed in the western area of the lake (Fig. 1) at a depth of 1.1 m and a sampling interval of 10 min. A high-pass cosine-Lanczos filter was used to filter the low-frequency signals from the lake-level series.

The wind data were obtained from 7 weather stations interconnected and distributed over the surface of water body (Fig. 1); records were taken for the dates of the two campaigns, and the sample interval was 10 min.

### Numerical model

The model used is a variant of the three-dimensional baroclinic HAMSOM model developed by Backhaus and collaborators at the Institut für Meereskunde, University of Hamburg^30,31^. The HAMSOM is a finite-difference, shallow-water model for simulating water levels and current velocities. Water levels are predicted by solving the generalized wave continuity equation based on the vertically integrated continuity equation:

1 $$\frac{\partial \zeta }{\partial t}+\frac{\partial U}{\partial x}+\frac{\partial V}{\partial y}=0$$

Where $U$ and $V$ are the depth-averaged velocities of the horizontal coordinates x and y, respectively; $\zeta$ is water surface elevation. The vertically integrated momentum equations may be written as follows:

2 $$\frac{\partial U}{\partial t}+\frac{U}{(H+\zeta )}\frac{\partial U}{\partial x}+\frac{V}{(H+\zeta )}\frac{\partial U}{\partial y}-fV=-\,g(H+\zeta )\frac{\partial \zeta }{\partial x}+{A}_{h}{\nabla }_{h}^{2}U+{\tau }_{s}^{x}-{\tau }_{b}^{x}$$

3 $$\frac{\partial V}{\partial t}+\frac{U}{(H+\zeta )}\frac{\partial V}{\partial x}+\frac{V}{(H+\zeta )}\frac{\partial V}{\partial y}+fU=-\,g(H+\zeta )\frac{\partial \zeta }{\partial y}+{A}_{h}{\nabla }_{h}^{2}V+{\tau }_{s}^{y}-{\tau }_{b}^{y}$$

where $H$ is the lake depth, $f$ is the Coriolis parameter, $g$ is acceleration due to gravity, ${A}_{h}$ is the horizontal eddy viscosity coefficient, ${\nabla }_{h}^{2}$ is the horizontal Laplacian operator, ${\tau }_{s}^{(x),(y)}$ is the wind stress, and ${\tau }_{b}^{(x),(y)}$ is the bottom stress friction. Those equations are subject to the kinematic and dynamic boundary conditions. Hence, kinematic boundary conditions for the free-surface $\zeta =\zeta (x,y,t)$ and bottom *z* = −*H*(*x*, *y*) are given as follows:

4 $${w}_{\zeta }=\frac{\partial \zeta }{\partial t}+{u}_{\zeta }\frac{\partial \zeta }{\partial x}+{v}_{\zeta }\frac{\partial \zeta }{\partial y}$$

5 $${w}_{-H}=-\,{u}_{\zeta }\frac{\partial H}{\partial x}-\,{v}_{\zeta }\frac{\partial H}{\partial y}$$

At the lateral boundaries, different boundary conditions are applied. For open boundaries, the water surface elevation $\zeta$ is given, and the change in the velocity component normal to the boundary vanishes; thus,

6 $$\frac{\partial {u}_{n}}{\partial {x}_{n}}=0$$

where ${u}_{n}$ and ${x}_{n}$ are the velocity component and coordinate normal to the boundary, respectively. At closed boundaries, the condition is as follows:

7 $${u}_{n}=0$$

The dynamic boundary conditions for the air-water interface (tangential wind stress) and the bottom stress friction are as follows:

8 $${\tau }_{s}^{(x),(y)}={\lambda }_{W}({W}^{(x)},{W}^{(y)})\sqrt{{W}^{(x)2}+{W}^{(y)2}}$$

9 $${\tau }_{b}^{(x),(y)}=\frac{r(u,v)}{{(H+\zeta )}^{2}}\sqrt{{U}^{2}+{V}^{2}}$$

where $\lambda {}_{W}=3.2\times {10}^{-6}$ is a proportionality constant, (*W*^(*x*)^, *W*^(*y*)^) are components of the wind velocity, and $r(u,v)=1.5\times {10}^{-3}$ is the friction bottom coefficient.

The model is written in a scheme of finite differences, and the type of mesh used is the Arakawa-C grid^32^. The numerical instabilities when simulating outflow in the numerical analysis of explicit time integration schemes in the HAMSOM model were avoided by ensuring the Courant-Friedrich-Lewy condition, which limits the maximum allowable discretization time steps^33–35^ and is expressed as follows:

10 $${\rm{\Delta }}t\le \frac{{\rm{\Delta }}{L}_{{\rm{\min }}}}{\sqrt{g{H}_{{\rm{\max }}}}}$$

where ${\rm{\Delta }}t$ is a time step and ${\rm{\Delta }}L$ is space step. The model was implemented with the bathymetry, wind speed, and direction data obtained from meterorological stations for the days of the campaigns. In addition, according to the Courant-Friedrich-Lewy condition, the ideal time step was determined for use in the simulations, which was 36.2 s, and the discretization of the mesh was ${\rm{\Delta }}x={\rm{\Delta }}y=300$ m. The model began with conditions of wind forcing for 28 days. The wind then stopped and allowed for free oscillations to be generated. After setting-up the model, it was validated by focusing on the longer-term evolution of the simulated series in order to determine whether undesired trends or other undesired effects were present. A comparison of the total daily averaged water level long time series was then performed.

In addition, we compared the measurements of the campaigns and performed modelling with the theoretical periods of seiches in a rectangular (Merian’s^36^ and a parabolic (approximation of Wilson^37^) basin with length L and constant water average depth H.The period of the k-th lake mode according to Merian’s formula^36^ is as follows:

11 $${T}_{k}=\frac{2L}{k\sqrt{g\,H}}$$

In the meantime, the period of the k-th lake mode according to approximation of Wilson^37^ is as follows:

12 $${T}_{k}=1.110\times [\frac{2L}{k\sqrt{g\,H}}]$$

High-frequency variability in the time series was extracted from the data and smoothed by a 60-min moving average. The time series were analysed in a time-frequency domain and filtered by high-pass cosine-Lanczos filter.

Power spectra were calculated using the fast Fourier transform to determine the periods of the lake seiches^38–40^. The number of degrees of freedom *df* was determined as follows: $df=2\alpha (2F+1)$, where $\alpha$ is the number of independent segments of realization in which the spectral estimates were averaged and *F* is the half-width of the filter used to average the periodograms.

## Results and Discussion

### Analysis of water level measurements

**Figure 2:**
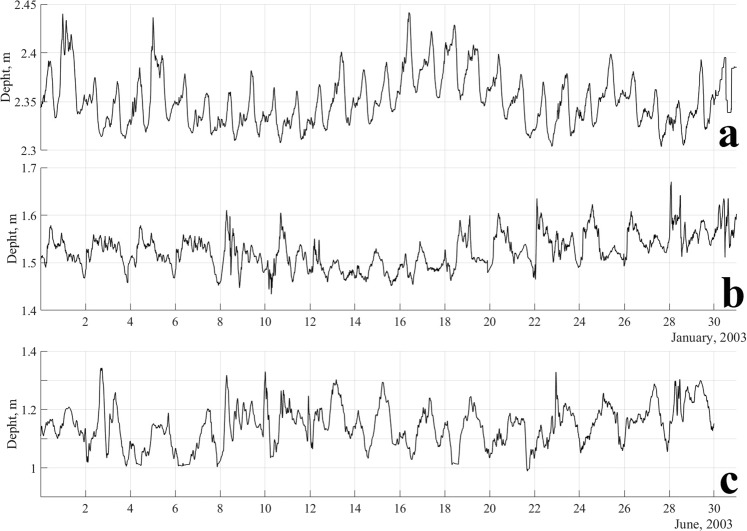
Oscillations of the water level in the lake at sites in the (**a**) northeast, (**b**) east and (**c**) west for the two sampling campaigns.

Lake-level measurements during the two campaigns are shown in Fig. 2, and these measurements may have high uncertainty due to contamination of the data by frequency aliasing and nonseiche oscillations as well as by water inflow and outflow. The most salient feature is the synoptic scale variability present at the three sampling sites, and it represents a response to fluctuations in meteorological characteristics^8^. The ascent and descent of the water level is asymmetric. The depression remains longer than the peak, this is an effect that is provided by the asymmetrical behaviour of the wind on the lake.

Level variations reach up to 30 cm for the sampling point in the west (Fig. 2c). In the case of the points to the east and northeast of the lake, the maximum level variations recorded are 17 and 12 cm, respectively (Fig. 2a,b). At the start, the maximum water level elevation coincides each time with a sudden change in air pressure, which is generally related to the development of the lake breeze. The magnitude of the wind of the daytime breeze reaches up to 6 m/s^23^.

**Figure 3:**
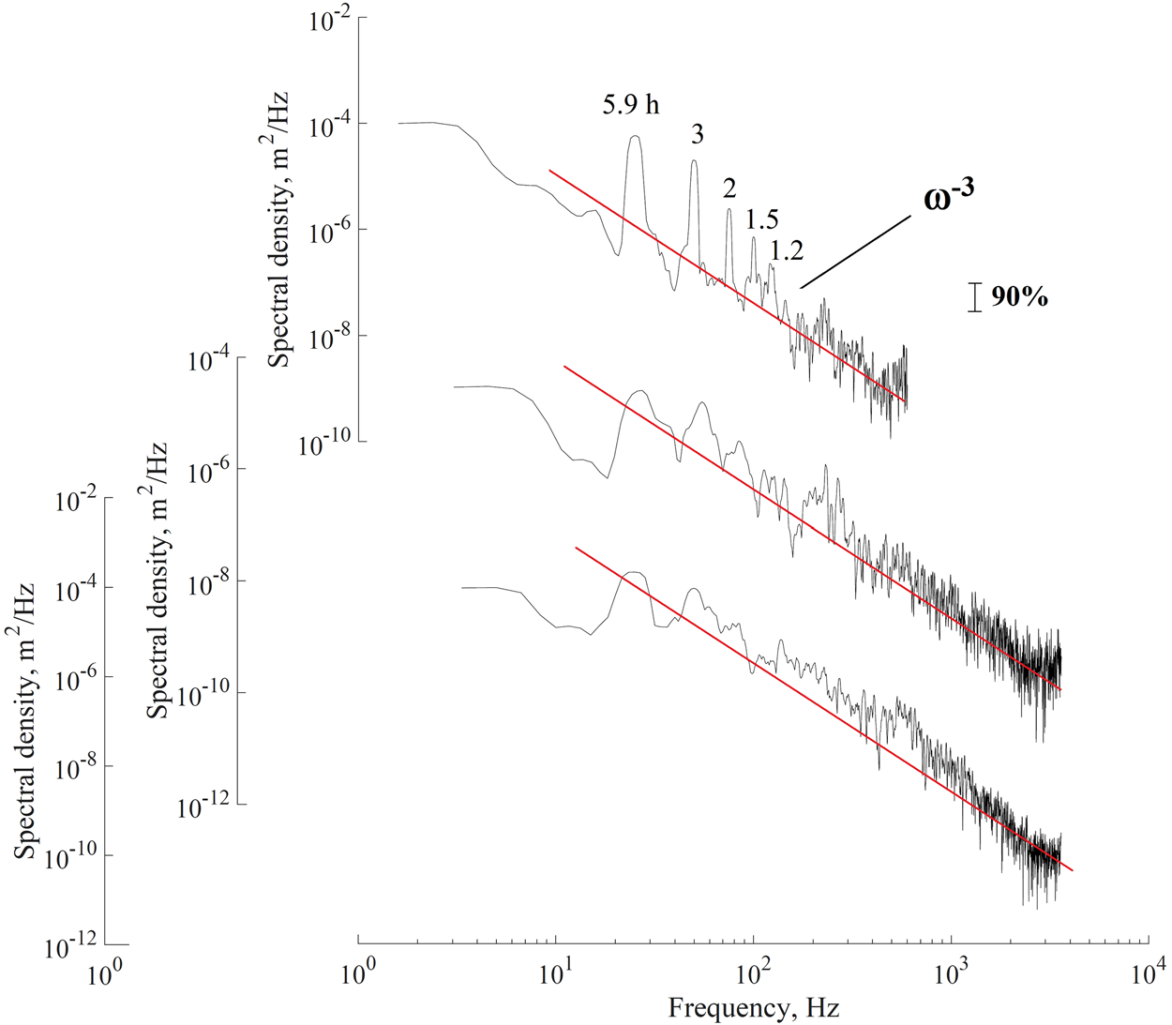
Frequency spectra of water level fluctuations in the lake at sites in the (**a**) northeast, (**b**) east, and (**c**) west for the two sampling campaigns. Arabic numerals show the period of the main peaks in the spectrum. The vertical line shows the 90% confidence interval with 10 degrees of freedom.

The frequencies of the seiches were determined by the fast Fourier transform and calculating the power spectrum. Figure 3 shows the normalized spectra of the lake water level measurement campaigns. Confidence intervals were calculated for all spectral estimates from standard techniques^38–40^.

The main peaks in the spectrum of the lake water level that are significant at or above the 90% confidence interval corresponding to the periods of 5.9, 3, 2, 1.5 and 1.2 h, 11, 5.5, 2.2, and 1.2 h, and 12, 5.9, 2.2, and 1.3 h for campaign measurements collected in the northeast, east, and west sites, respectively. Also, is observed that for band the spectral falloff rate with frequency ω tends to ω^−3^.

Level oscillations that have a period of 5.9 probably represent the seiches that propagate from west to east, because the lake is oval in shape, so the axes are different sizes, and this direction corresponds to the direction of the major axis. In contrast, the seiches that propagate from north to south (minor axis) belong to the other mode of oscillation.

### Numerical modelling of seiches (free oscillations)

**Figure 4:**
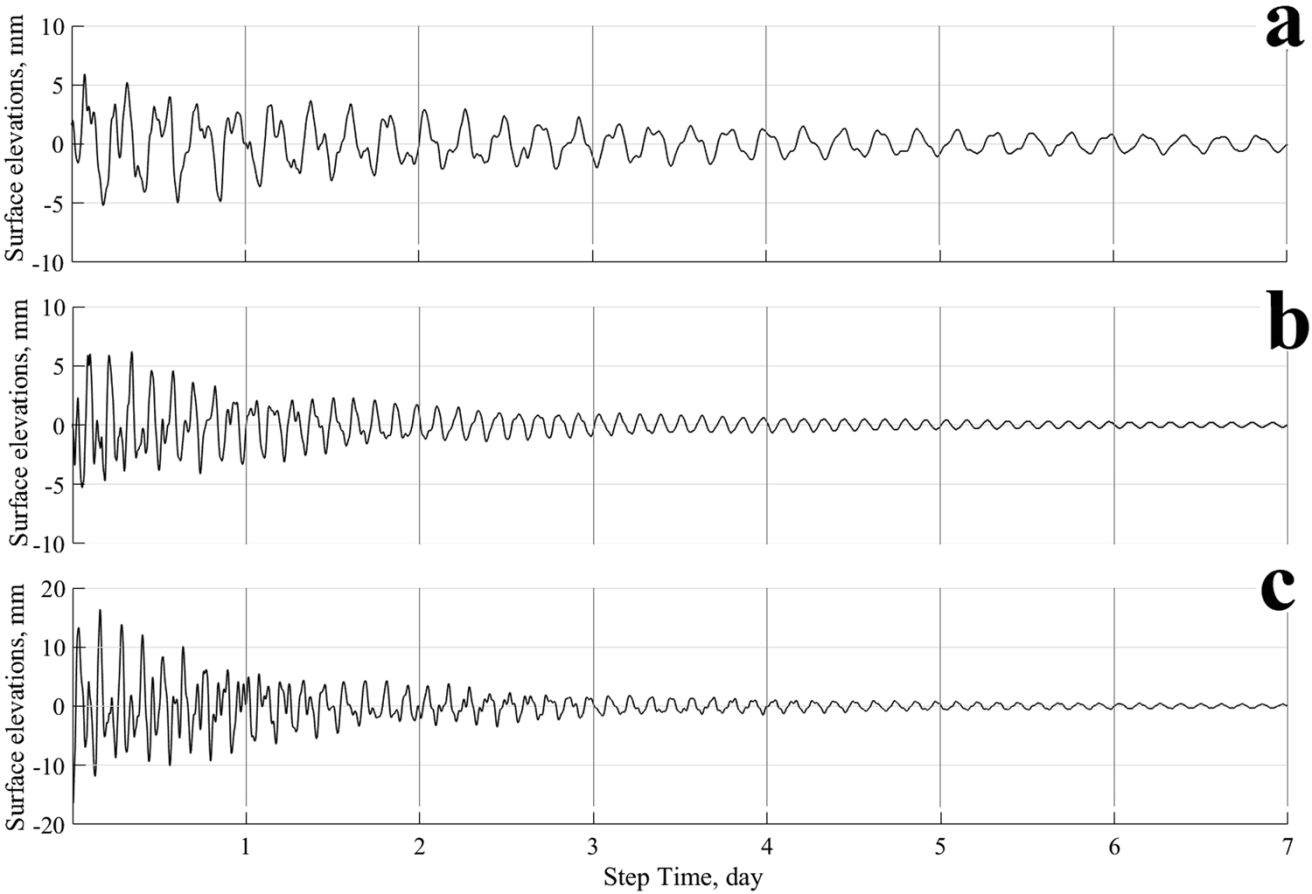
Modelling of surface water elevations at the measurement sites (**a**) northeast, (**b**) east, and (**c**) west.

In the simulation, three control points were adjusted to the HAMSOM model, and they coincide with the measurement sites in the lake. This simulation was performed to obtain comparable information on variations of the lake water level. The results of the last 7 days of simulation of the water level are presented here, with a discretization of 36 s for each recording point (Fig. 4). Level oscillations are higher at the western recording point and reach a maximum value of 17 mm, while at the other two recording points, they hardly exceed 5 mm.

**Figure 5:**
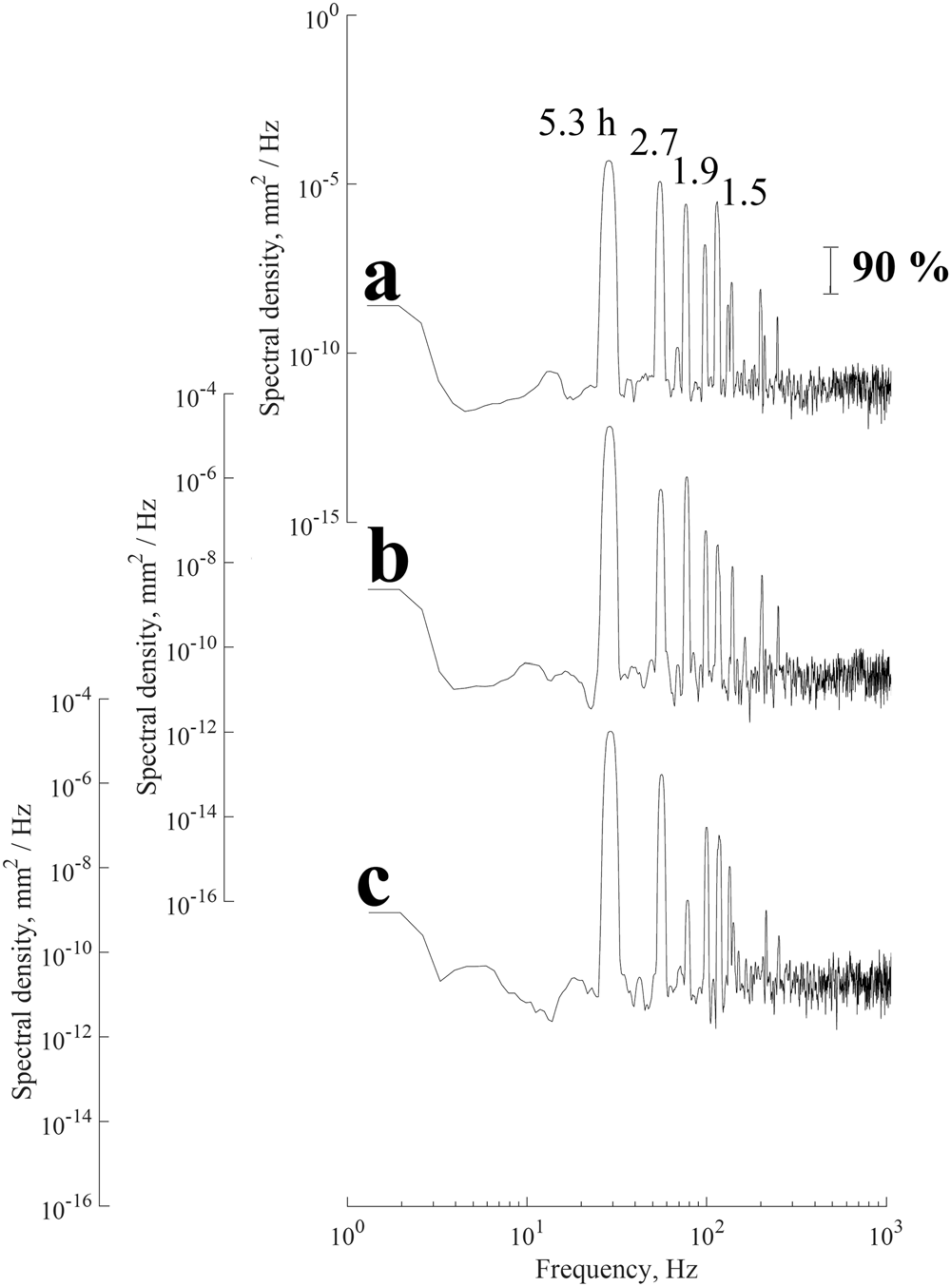
Frequency spectra of the modelled water level fluctuations in the lake at sites in the (**a**) northeast, (**b**) east, and (**c**) west. Arabic numerals show the periods of the main peaks in the spectrum. The vertical line shows the 90% confidence interval with 10 degrees of freedom.

Figure 5 shows the normalized spectrum of the high-frequency signals, and it indicates that there are free seiches with periods of 5.2, 2.7, 1.9 and 1.5 h in the lake, which are within the established confidence level. The highest energetic peaks are those of the first node seiches in the longitudinal direction due to the higher frictional resistance in the shallow water.

The spatial structure of the high-frequency seiches with periods of less than 1.5 h is very complicated due to the strong effect of the small-scale features of the basin geometry and bottom topography, which explains why level oscillations are not identical for the complete lake.

**Figure 6:**
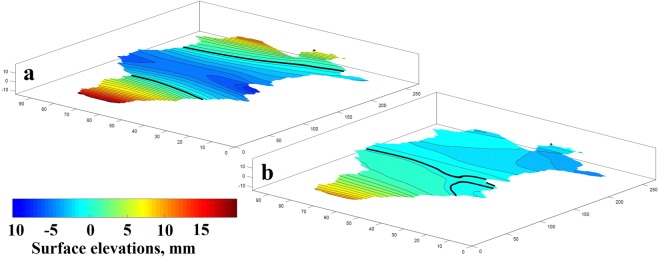
Level surfaces of seiche modes with the periods of (**a**) T = 5.2 h and (**b**) T = 2.7 h modelled for the west site.

Figure 6a shows the deviations of the lake level for the high-frequency seiches (the period is 5.2 h) in which the evolution of two transverse nodal lines are presented and show that the maximum amplitudes occur in the shallow western part of the lake. The level deviations for the seiche with a period of 2.7 h presents the evolution of a transverse nodal line that crosses the entire width of the lake as well as a small longitudinal node close to the south-central coastline.

**Figure 7:**
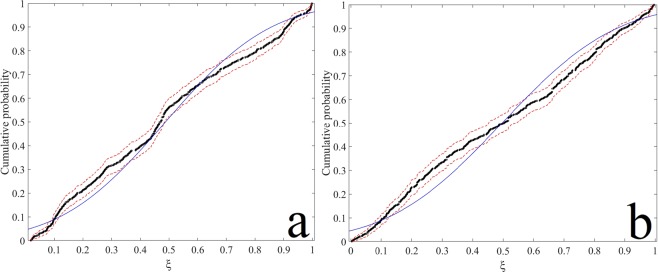
Cumulative probabilities of (**a**) measurements water level (dots black) (**b**) results of the free oscillations obtained by the HAMSOM model (dots black). The line (blue) is the theoretical prediction and the dashed lines (red) are the 90% confidence limits.

The uncertainty of the water level measurements and the results of the free oscillations obtained by the HAMSOM model have been evaluated by the Monte Carlo simulation described by Coleman and Steele^41^, Longo, *et al*.^42^, Di Federico, *et al*.^43^. Figure 7 shows the cumulative probabilities from the Monte-Carlo simulation and water level measurements and free oscillations obtained by the HAMSOM model. This is due to the fact that the model do not generate low frequency signals. However, for the central part of the cumulative probabilities coincides well with the measurements and modelled. However, the cumulative probabilities may be noticeably different from the true one, but the bounds accurately contain the true cumulative probabilities.

### Theoretical calculation of seiche periods

The estimates of lake oscillation modes obtained from Merian’s formula (Eq. 11) and approximation of Wilson (Eq. 12) are presented in Table 1. The periods of the first five modes are consistent what was observed and modelled in the northeastern lake area. The seiche modes measured on the east and west shores of the lake are consistent with the first, second, and fourth oscillation modes (Merian’s formula). However, the other mode measurements do not fit with their respective theoretical calculations, which is likely due to the irregularities in bathymetry because a rectangular lake with a constant depth of 6 m is assumed for these calculations. Likewise, the estimations from Wilson’s approximation for a parabolic-shaped basin, showed that lake oscillations match for all measurements modes.

**Table 1:** Periods of free oscillation modes theoretical in Lake Chapala.

Node	Period/h Merian’s formula 36	Period/h approximation of 37
1	5.4	6.0
2	2.7	3.0
3	1.8	2.0
4	1.4	1.5
5	1.1	1.2

The modelling results provide information on the spatial distribution of seiche features but not on the absolute magnitude of water level oscillations. Previous studies Avalos-Cueva, *et al*.^22^ and Filonov^8^ have shown that the currents produced by the lake breeze front can vary significantly and spatially depending on the characteristics of the wind or other disturbances.

Therefore, measuring level oscillations in different areas of the lake is appropriate for analysing the periods in specific areas. However, discrete regularities are inherent even in waves with small periods. Oscillations are often observed in the central part and on the east and west coasts of the lake. Therefore, the oscillations of high-frequency seiches can be considered to occur throughout the lake.

### Periods of seiches at different isobaths

The greatest depth of the lake is nearly 11 m. However, the interannual fluctuations of Lake Chapala can be as great as 6 m in extreme years^29^; hence, the periods of seiches can vary (increasing or decreasing). The model was adapted to six different storage levels of the lake from the 90 m isobath (corresponding to an average depth of 1 m) to the 95 m isobath (corresponding to an average depth of 8 m). The model was run with the same considerations detailed in the methodology.

**Figure 8:**
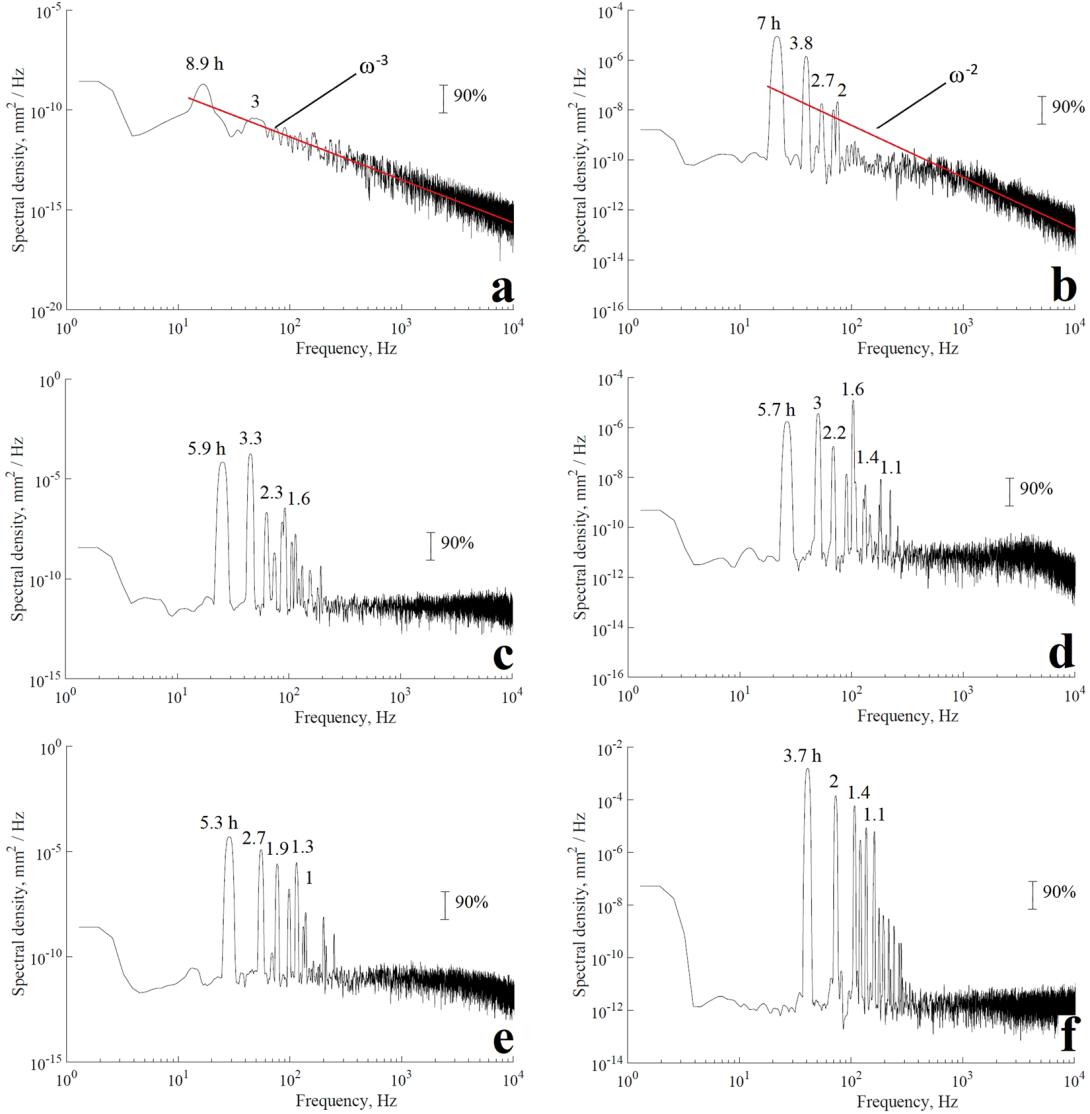
Frequency spectra of the modelled water level fluctuations in the lake at the west site from the different water storage levels (from isobaths 90 to 95 m). Arabic numerals show the period of the main peaks in the spectrum. The vertical line shows the 90% confidence interval with 10 degrees of freedom.

Figure 8 shows the normalized spectra of the water level series for the isobaths at 90 to 95 m. The modal structure of seiches resulting from the decrease in storage isobaths shows a cascade effect in the periods of the main modes of oscillation. The frequencies of the main peaks in the 90 m isobath have periods of 8.9 and 3 h for the first two modes in addition to other frequencies outside the confidence interval (Fig. 8a). The inclined red line in this figure shows the ω^−3^ function again, as in the spectra of the lake water level measurement; there is a good agreement between the slope of the spectrum of water level oscillations and rate of attenuation of modelling spectrum in the frequency interval for the 90 m isobath.

In the isobaths from 91 to 95 m, the first four oscillation modes that were recorded in the spectra of the measurements in the lake are again observed (Fig. 8b–f). The first mode shows periods between 7 and 3.7 h with ascending isobaths (Table 2). The difference between the periods does not exceed 2 h for the following oscillation modes. In Fig. 8b, the red line showed an inclination of spectral density of ω^−2^ for the isobath of 91 m, it is due to the fact that the data-register point is near the coast and the signal is contaminated with low frequency noise.

**Table 2:** Period of the first longitudinal mode as a function of the isobaths.

Isobaths, m	Period/h
90	8.9
91	7
92	5.9
93	5.7
94	5.3
95	3.7

The periods recorded for the first oscillation mode in the different isobaths were adjusted using the minimum squares method. The fitting equation is defined as follows:

13 $${T}_{1}=0.0768{L}_{c}^{2}-15.1{L}_{c}+745.58$$

where ${T}_{1}$ is the first oscillation mode period (in hours) and ${L}_{c}$ is the isobath value (in meters).

**Figure 9:**
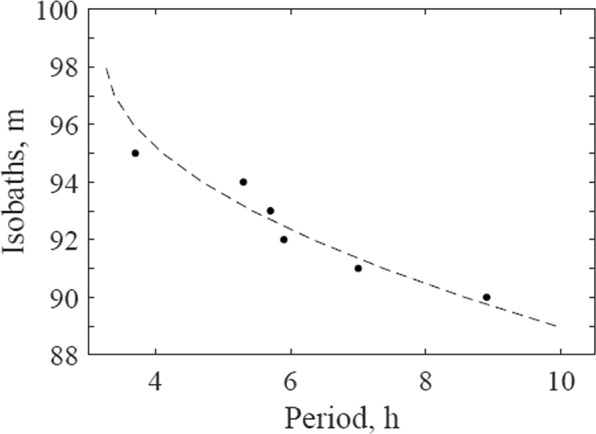
Points refer to the first mode seiche periods obtained from modelling using the different storage isobaths for the lake, and the solid line is the fitting curve obtained by Eq. 13.

Figure 9 shows the adjustment curve for the first mode seiche periods. The correlation coefficient between the curve and the periods of the first mode (the isobaths as a function of period) is 0.93.

## Conclusions

The research results provide the spatial distribution of lake water level oscillations for seiche periods. Furthermore, the five modes theoretically defined from Merian^36^ and Wilson^37^ formulas are observable in the spectra of the lake water level fluctuations measured and modelled for the three study sites. The modes calculated from Wilson’s approximation for a parabolic-shaped basin matched the measurements almost exactly.

The oscillations in lake water levels measured in the lake for the first five seiche modes were characterized by periods of 5.9, 3, 2, 1.5 and 1.2 h, whereas the modelled water level presented periods of 5.2, 2.7, 1.9, 1.5 and 1.2 h for the same five modes. Nonetheless, as has been observed in other water bodies, the friction produced by the bottom is an important factor that controls the dissipation of energy, allowing oscillations to cease in less or more time. That is to say, if a lake presents a topography with soft unevennesses, the friction is reduced, allowing the oscillations to remain for a longer time. As a consequence, if forced disturbances are made at the resonance frequency, the oscillations can be significant and will manifest for several hours. The difference presented between the model and the measurements for the first mode is of less than one hour, this is because the model used phases-averaged and does not include all these sources or sink of energy and momentum. However, the periods of the seiches obtained from the measurements and modelling exhibit high correspondence.

The lake-level water elevations generated by the model showed two transverse nodal lines for the first mode, with maximum heights along the east and west coasts reaching 17 and 14 mm, respectively. The second mode corresponding to the 2.7 h period displayed two nodal lines in the central part of the lake (transverse and longitudinal). The first completely crosses the lake from north to south, while the second is located in a smaller south-central area of the lake.

Finally, we can conclude that the HAMSOM model is a useful approximation for studying seiches in Lake Chapala. In addition, the presented results allowed us to evaluate the seiches’ modes at different levels of storage that could be present in Lake Chapala. In conclusion, it has been shown that the periods of the first seiche modes decrease as the lake (isobath) storage level decreases. The difference in period between the 90 to 95 m isobath of the first seiche mode has reached 5.2 h.

This paper provides the first limnological approach to understanding free periodic oscillations in Mexican lakes. Theoretical calculations of the seiches in the lake require experimental calibration, which the authors plan to perform in the near future. It is also necessary to carry out a study with a three-dimensional model that allows for the inclusion of several layers, energy sources, and turbulence.

## Data Availability

The datasets generated during and/or analysed during the current study are available from the corresponding author on reasonable request.
